# Hepatitis C Viral Entry Inhibitors Prolong Viral Suppression by Replication Inhibitors in Persistently-Infected Huh7 Cultures

**DOI:** 10.1371/journal.pone.0065273

**Published:** 2013-06-03

**Authors:** Caroline O. Bush, Andrew E. Greenstein, William E. Delaney, Rudolf K. F. Beran

**Affiliations:** Gilead Sciences, Inc., Biology Department, Foster City, California, United States of America; University of North Carolina School of Medicine, United States of America

## Abstract

Efforts to treat HCV patients are focused on developing antiviral combinations that lead to the eradication of infection. Thus, it is important to identify optimal combinations from the various viral inhibitor classes. Based on viral dynamic models, HCV entry inhibitors are predicted to reduce viral load in a monophasic manner reflecting the slow death rate of infected hepatocytes (t_1/2_ = 2–70 days) and the protection of naïve, un-infected cells from HCV infection. In contrast, replication inhibitors are predicted to reduce viral load in a biphasic manner. The initial rapid reduction phase is due to the inhibition of virus production and elimination of plasma virus (t_1/2_∼3 hours). The second, slower reduction phase results from the elimination of infected hepatocytes. Here we sought to compare the ability of HCV entry and replication inhibitors as well as combinations thereof to reduce HCV infection in persistently-infected Huh7 cells. Treatment with 5×EC_50_ of entry inhibitors anti-CD81 Ab or EI-1 resulted in modest (≤1 log_10_ RNA copies/ml), monophasic declines in viral levels during 3 weeks of treatment. In contrast, treatment with 5×EC_50_ of the replication inhibitors BILN-2016 or BMS-790052 reduced extracellular virus levels more potently (∼2 log_10_ RNA copies/ml) over time in a biphasic manner. However, this was followed by a slow rise to steady-state virus levels due to the emergence of resistance mutations. Combining an entry inhibitor with a replication inhibitor did not substantially enhance the rate of virus reduction. However, entry/replication inhibitor and replication/replication inhibitor combinations reduced viral levels further than monotherapies (up to 3 log_10_ RNA copies/ml) and prolonged this reduction relative to monotherapies. Our results demonstrated that HCV entry inhibitors combined with replication inhibitors can prolong antiviral suppression, likely due to the delay of viral resistance emergence.

## Introduction

Researchers are actively working to develop inhibitors of several stages of the hepatitis C viral (HCV) lifecycle including entry, replication, and assembly [1]–[5]. A curative antiviral therapy for HCV-infected patients will likely be comprised of a combination of two or more distinct viral inhibitors. An optimal HCV inhibitor combination will prevent the virus from acquiring resistance mutations and lead to eradication of the virus from the patient.

In recent years, significant progress has been made toward understanding HCV entry [6], [7] and developing inhibitors of this process [2], [7]–[11]. HCV entry is initiated by the attachment of viral envelope proteins (E1 and E2) to glycosaminoglycans [12] followed by a post-attachment stage which includes specific binding to cellular receptors and subsequent uptake into the cell. The five cellular receptors known to be utilized by HCV are the tetraspanin protein CD81 [13], scavenger receptor class B member 1 [14], the Niemann-Pick C1-like 1 cholesterol absorption receptor [7], claudin 1 [15], and occludin [16], [17]. In addition, the tyrosine kinases epidermal growth factor receptor and ephrin receptor A2 are thought to act as HCV entry co-factors by modulating the interaction between CD81 and claudin 1 [18]. After receptor binding, HCV undergoes clathrin-mediated endocytosis and fusion between the virion envelope and the endosomal membrane [17], [19]. Anti-CD81 antibody (Ab) has been used to successfully block HCV binding of the CD81 receptor and viral uptake into the cell [20], [21]. In addition, Entry Inhibitor-1 (EI-1) is a small molecule that inhibits HCV genotype 1a and 1b entry during the post-attachment phase, likely during the fusion step [2].

Though there has been progress in understanding HCV entry and developing entry inhibitors, HCV viral dynamic models predict that entry inhibitors will have a slow and modest antiviral activity as monotherapies in chronically-infected patients [22]. These models predict that entry inhibitors would reduce viral load in a monophasic manner reflecting the slow death rate of infected hepatocytes *in vivo* (t_1/2_ = 2–70 days) and the protection of naïve uninfected cells from HCV infection. In contrast, replication inhibitors are predicted to reduce viral load in a biphasic manner. The initial rapid reduction phase is due to the inhibition of virus production and elimination of plasma virus (t_1/2_ ∼3 hours). The second, slower reduction phase results from the elimination of infected hepatocytes [22]. However, for many classes of replication inhibitors, monotherapy leads to the rapid emergence of viral resistance mutations [23]–[25]. Combining two replication inhibitors with different targets or a replication inhibitor with an entry inhibitor would theoretically impact the emergence of resistance by increasing the number of viral mutations required to break through therapy. Because some mutations are less likely to emerge than others [24] and because some mutations reduce viral fitness [23], [25], an optimal combination of inhibitors must be investigated experimentally.

Here we sought to determine if HCV entry inhibitors alone can reduce viral levels in persistently-infected Huh7 cultures. Also we sought to determine if HCV entry inhibitors combined with HCV replication inhibitors can provide a greater reduction in viral levels than either monotherapy in persistently-infected cultures. Finally, we wanted to determine if an entry/replication inhibitor combination could prolong reductions in viral levels relative to replication inhibitor monotherapy. To enable these studies, we first demonstrated that persistently-infected Huh7 cell cultures can be established using tissue-culture adapted HCV and used as a model system to monitor extracellular virus levels during antiviral treatment. Using these persistently-infected cell cultures, we observed that entry and replication inhibitor monotherapies fit the model previously proposed for viral load reduction during short-term treatment. Entry inhibitor monotherapy caused a slow, monophasic reduction in viral levels, while replication inhibitor monotherapy caused a rapid, biphasic reduction. This suggests that entry inhibitors will only have a modest impact on serum HCV RNA levels in chronically-infected patients who have minimal viral spreading. However, our results also demonstrated that the combination of an entry plus replication inhibitor can prolong antiviral suppression, likely due to the delay of viral resistance emergence.

## Materials and Methods

### Cell Culture

Huh7-Lunet-CD81 [26] cells were propagated in Dulbecco’s Modified Eagle Medium (D-MEM) with GlutaMAX™-I (Invitrogen, Carlsbad, CA) supplemented with 10% FBS (HyClone, Loglan, UT), 0.1 mM non-essential amino acids (Invitrogen). Cells were maintained in humidified incubators at 37°C and 5% CO_2_.

### Antiviral Compounds and Antibodies

The HCV NS3–4A protease inhibitor BILN-2061 was purchased from Acme Biosciences (Belmont, CA). The HCV NS5A inhibitor BMS-790052 was purchased from Selleck Chemicals (Houston, TX). The mouse monoclonal anti-human CD81 antibody JS-81 was purchased from BD Biosciences (San Jose, CA). The HCV entry inhibitor-1 (EI-1) was purchased from ChemBridge Corporation (San Diego, CA).

### Antiviral and Cytotoxicity Assays

Huh7-Lunet-CD81 cells [26] were seeded in white clear bottom 96 well plates at a density of 5000 cells per well. After overnight incubation, three-fold serial compound dilutions were prepared in DMSO, diluted 250-fold into a DMEM viral stock, and added to the cells in 96-well plates in a final volume of 100 µl. Final compound concentrations typically ranged from 2.5 to 50,000 nM and the MOI was 0.3 or greater. Anti-CD81-monoclonal antibody was serially diluted in DMEM yielding final concentrations from 0.25 ng/ml to 5000 ng/ml. Following 3 days of incubation, NS3–4A protease activity was used to quantify intracellular HCV replication levels as described below. The resulting data was fit to the Hill equation using SigmaPlot (Systat) to calculate EC_50_ values. For cytotoxicity assays, cells were incubated with compounds as described for antiviral assays, with the exception that no virus was added. Following 3 days of incubation, intracellular ATP levels were measured using a Cell-Titer Glo kit according to the manufacturer’s instructions (Promega, Madison, WI). The resulting data was fit to the Hill equation using SigmaPlot to calculate CC_50_ values.

### Intracellular NS3–4A Protease Activity

NS3–4A protease activity was used to monitor intracellular HCV replication levels and was measured using a europium labeled NS3–4A protease substrate as described previously [27] with slight modifications. In brief, media was removed from virus-infected cells and replaced with 50 µl of a lysis/NS3–4A substrate solution containing 1× lysis buffer (Promega, Madison, WI); 150 mM NaCl, and 150 nM NS3–4A europium substrate (AnaSpec, Freemont, CA) in deionized water. Time-resolved fluorescence was measured for 10 cycles using a VICTOR^3^™ V Multilabel Counter (Perkin Elmer, Waltham, MA).

### Indirect Immunofluorescence of HCV Infected Cells

Infected cells were grown in 96-well plates and fixed with 50 µl/well glacial methanol-acetone (1∶1) at room temperature for 20 minutes. Cells were then washed three times with phosphate-buffered saline (PBS). Immunostaining of NS5A was performed by using a mouse monoclonal antibody (9E10; Apath, Brooklyn, NY) at a dilution of 1∶4000 in PBS with 3% bovine serum albumin (BSA) for 1 hour at room temperature. After two washes with PBS, bound primary antibodies were detected by using a mouse antibody conjugated to Alexa-Fluor 555 (Life Technologies, Foster City, CA) at a dilution of 1∶3,000 in PBS containing 3% BSA for 20 minutes in the dark at 4°C. DNA was stained with 4,6-diamidino-2-phenylindole dihydrochloride (Molecular Probes, Madison, WI) for 10 minutes at 4°C in the dark. Finally, cells were washed three times with PBS and imaged by using a Zeiss microscope with fluorescence capabilities (Thornwood, NY). To guide infected *vs*. non-infected cell scoring, the percentage of infected cells was quantified using an ImageXpress Micro (Molecular Devices) (Sunnyvale, CA) where feasible. After images were acquired with a 10× objective, automated infectivity determination was conducted by first identifying each cell nucleus and then determining if the Alexa-Fluor 555 coincident with that nucleus was above (infected) or below (uninfected) a heuristically-determined threshold.

### HCV Persistently-infected Cultures

**Figure 1:**
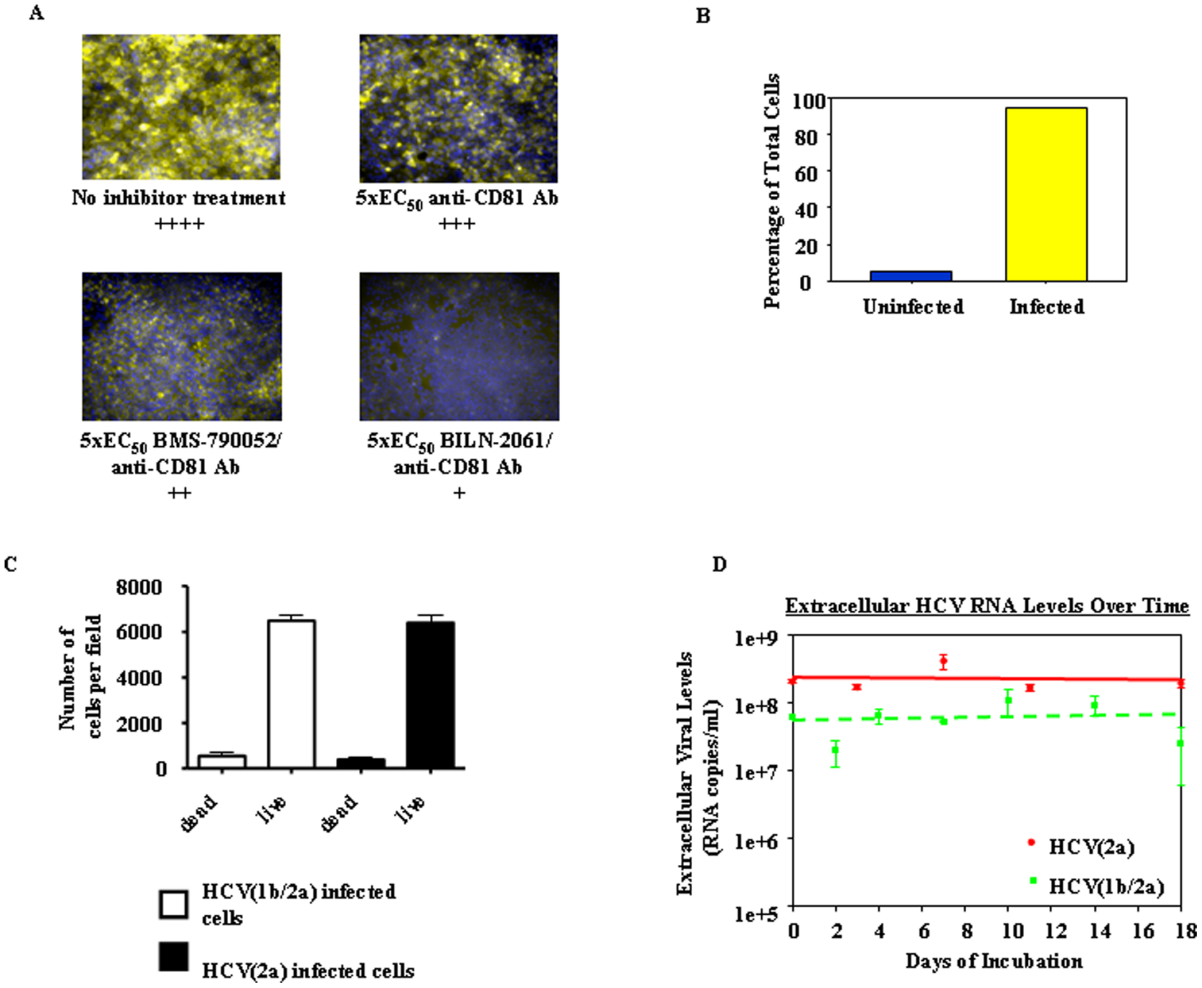
Characterization of HCV persistently-infected cultures. (A) Examples of the level of HCV(1b/2a)-infected cells *vs*. uninfected cells after 21 days with or without replication inhibitor/entry inhibitor combination therapy. The yellow cells are infected (*i.e.* stained with anti-NS5A Ab as described in the Materials and Methods) and the blue cells are uninfected. The pluses signify relative quantifications of the percentage of infected cells in each culture (see Materials and Methods). (B) Bar graph depicting quantification of infected *vs*. uninfected cells in the untreated HCV persistently-infected culture shown in Figure. 1A (see Materials and Methods). (C) Viability of HCV persistently-infected cells after 25 days of incubation (see Materials and Methods). (D) Extracellular HCV levels (log_10_ RNA copies/ml) during an 18-day time course initiated 7 days post infection (average of 3 assays) (HCV(2a) levels (solid circles), HCV(1b/2a) levels (solid squares, dashed line)).

HCV persistently-infected cultures were established using methods adapted from Sainz and Chisari (2006) [28] and Beran *et al*., (2012) [29]. Briefly, Huh7-Lunet-CD81 cells [26] were seeded in 12-well plates at a density of 50,000 cells/well. The plates were incubated over-night at 37°C and subsequently DMSO was added to 1% final concentration. After 3 days, the cell cultures became confluent and infectious HCV was added to each well. Infectious HCV(2a) with three adaptive mutations (the Min3 virus previously described) [30] or HCV(1b/2a) (a chimeric virus expressing the genotype 1b structural genes and the genotype 2a non-structural genes and 6 adaptive mutations as previously described [26]) was then added to each well at an MOI of 5. Infection was permitted to spread for seven days until the cultures were ∼95% infected (determined by NS5A immunofluorescence [30] quantified on an ImageXpress Micro system (Molecular Devices, Sunnyvale, CA)). Subsequently, compounds of interest were added at concentrations equal to 5×EC_50_. 500 µl aliquots of the extracellular medium were saved at various days after drug addition and stored at −80°C for future analyses (see below). Media containing compounds were refreshed after taking each time point. Typically, media samples were collected and compound media were refreshed on days 0, 1, 2, 4, 7, 10, 14, 18, and 21. On the final day of the time courses, the cell cultures were fixed with ice-cold methanol for 15 minutes for subsequent indirect immunofluorescence as described above. The percentage of HCV-infected cells in each culture were estimated by viewing them under a fluorescence microscope as described above. Additionally, the percentage of infected cells in the cultures after 3 weeks of inhibitor treatment was quantified using the ImageXpress Micro. Because the inhibitor-treated confluent cultures had cells growing on top of cells, absolute accuracy of the automated infectivity quantification was limited. Thus, we presented estimated differences in the percentage of infected cells using a series of pluses to show relative differences in the percentages of infected cells. Examples of the difference in the number of infected cells for “++++”, “+++”, “++”, and “+” cultures are shown in figure 1A. These scores were guided both by automated infectivity quantification and manual observation with a fluorescence microscope.

### Quantifying Viable Cells in the HCV Persistently-infected Cultures

Cell cultures were stained with calcein to recognize living cells and with propidium iodide to recognize dead cells according to the manufacturer’s protocol in a Cellomics Cell Viability HCS Reagent Kit (Thermo Scientific, Rockford, IL). An ImageXpress Micro system was used to quantify the number of living and dead cells. Alternatively, a Cell Titer Glo Kit (Promega, Madison, WI) was used to measure ATP levels in cellular lysates as a means to quantify cellular viability.

### RNA Purification and Quantification

Stored aliquots of media collected from infected cells were thawed and extracellular viral RNA was isolated. Extracellular viral RNA isolation was performed using a QIAamp Viral RNA Mini Kit (Qiagen, Valencia, CA) according to the manufacturer’s protocol. A QuantiFast Probe RT-PCR Kit (Qiagen) was used according to the manufacturer’s protocol to quantify the RNA levels in a 96-well format on an Applied Biosystems 7300 Real Time PCR System (Carlsbad, CA). The DNA primers used were synthesized by Integrated DNA Technologies (Coralville, IA). These primers amplified the HCV(2a) NS3 gene and were designated NS3 2a+ (5′ cgg tcc gag tac atc tgc gtg ac (FAM) g 3′) and NS3 2a- (5′cac gga gct ggc aac aag act 3′). The detection limit with this assay was observed to be 1e^3^ RNA copies/ml. Also, it should be noted that the media was changed after each time point was collected. Thus, the extracellular HCV RNA measured each time represented *de novo* HCV RNA that had been released since the previous time points.

### Clonal Sequencing

Clonal sequencing was performed to identify resistance mutations at the end of the HCV persistently-infected time-course experiments. Briefly, extracellular viral RNA were isolated from samples collected on the final day of the time courses as described above. Viral RNA were reverse transcribed using Superscript III (Life Technologies, Carlsbad, CA) according to the manufacturer’s protocol. Resulting cDNAs were amplified by PCR using primers specific to the E2(1b), E2(2a), NS3(2a), or NS5A(2a) genes. The DNA primers used were E2-1b start (5′ gga acc tat gtg aca ggg 3′), E2-1b end (5′ ctc agc ttg agc tat cag cag 3′), E2-2a start (5′ cgc acc cat act gtt ggg ggt 3′), E2-2a end (5′ ttc ggc ctc gcc caa caa gat 3′), NS3-2a start (5′ ctc gct ccc atc act gct tat 3′), NS3-2a end (5′ cat gac ctc aag gtc agc ttg 3′), NS5A-2a start (5′ tgc tcc gga tcc tgg ctc c 3′), and NS5A-2a end (5′ gca cac ggt ggt atc gtc ctc 3′). PCR reactions were performed using ExTaq polymerase (Thermo Fisher Scientific, Waltham, MA). PCR products were purified using a Qiagen PCR Clean-Up Kit (Qiagen, Valencia, CA), and were subsequently ligated into a TOPO TA pCR4 cloning vector (Life Technologies) according to the manufacturers’ protocols. Ligation products were transformed into TOP10 frozen competent cells (Life Technologies) and clones were selected on LB-carbenicillin plates according to the manufacturer’s protocol. Plasmid DNA was prepared from the selected clones using a Qiagen MiniPrep Kit according to the manufacturer’s protocol and DNA sequencing was performed by Elim BioPharm (Hayward, CA).

## Results

### Long-term HCV Persistently-infected Cultures can be Established

To determine whether HCV entry inhibitors are able to reduce extracellular viral levels in persistently-infected cells, we first needed to establish long-term HCV persistently-infected cell cultures. Initial attempts to establish long-term HCV persistently-infected cell cultures failed because the majority of the infected cells died after reaching confluence (data not shown). Thus, based on previously described methods [28], [29], [31], we added DMSO to 1% final concentration to Huh7-Lunet CD81 cells growing on 12-well plates. Within 3–4 days, a confluent cellular layer formed in each well and we infected the cultures using high-titer genotype 2a HCV or genotype 1b/2a HCV stocks. Infections were allowed to spread throughout the cultures for 7 days until approximately 95% of cells were infected as determined by NS5A staining (Fig. 1A “no inhibitor treatment case” and 1B) (see Materials and Methods). We demonstrated that these cultures contained primarily live cells even after 25 days of incubation by staining these cultures with calcein and propidium iodide (Fig. 1C) (see Materials and Methods). Uninfected cell cultures treated with 1% DMSO exhibited a similar ratio of live *vs*. dead cells over the same time frame (data not shown). The level of HCV infection in the cultures remained relatively stable during an 18-day time course initiated 7 days post-infection as observed through following viral RNA levels in the extracellular media (RNA copies/ml) (Fig. 1D). It should be noted that after extracellular media was collected at each time point, the media was refreshed. Thus, the HCV RNA levels measured represented *de novo* release of HCV RNA from the cells since the previous time point. Also, the DMSO-treated cultures remained highly infected at the end of the time course as observed through NS5A staining. However, because cells continued to slowly grow on top of cells over time based upon microscopy and intracellular ATP measurements (see Materials and Methods) (data not shown), it was not possible to accurately quantify the percentage of infected cells after 3 weeks of incubation. Rather, relative quantifications of the percentage of infected cells are presented throughout this work using a series of pluses (see Materials and Methods and Fig. 1A). Based upon the above results, HCV persistently-infected cultures can be established and remain stable for several weeks.

### HCV Entry Inhibitor Monotherapy Slowly Reduced Viral Levels Over Time in Persistently-infected Cultures

**Figure 2:**
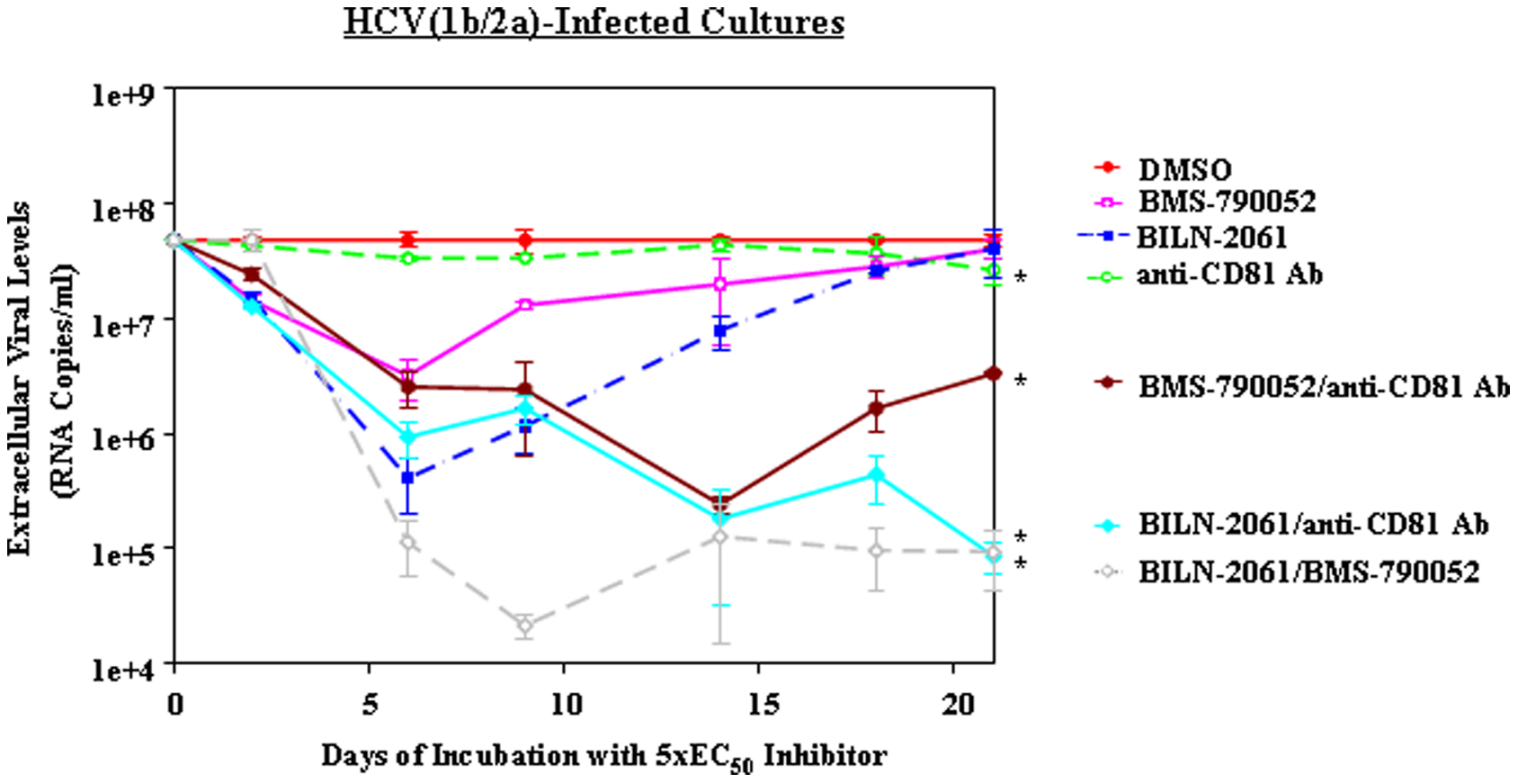
Reduction of HCV(1b/2a) levels by anti-CD81 Ab alone and in combination with BILN-2061 or BMS-790052. HCV(1b/2a) persistently-infected cell cultures were treated with 5×EC_50_ concentrations of the indicated HCV inhibitors for 21 days (see Table 1 and Materials and Methods). HCV levels were normalized relative to the level of the DMSO control at each time point. This data is the average of 3 assays. Error bars represent standard deviation. Asterisks indicate statistically significant differences at day 21 from the DMSO day 21 time point (*t* test P≤0.05). (A) DMSO (solid circles and solid line), anti-CD81 Ab (pierced circles and dashed line), BILN-2061 (solid squares and dashed line), BMS-790052 (pierced squares and solid line), BILN-2061/anti-CD81 Ab (solid diamonds and solid line), BMS-790052/anti-CD81 Ab (solid hexagons and solid line), and BILN 2061/BMS-790052 (pierced diamonds and dashed line).

**Figure 3:**
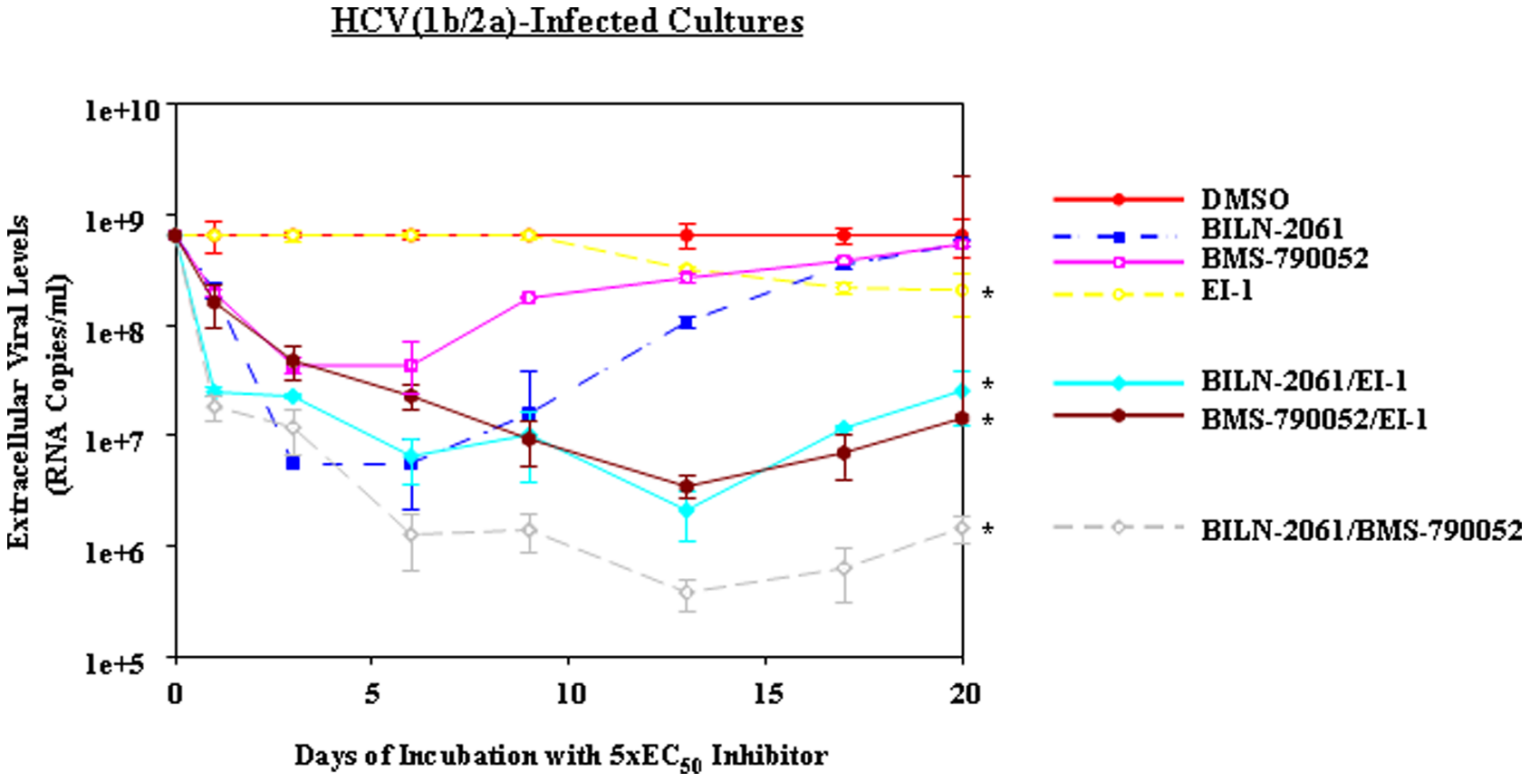
Reduction of HCV(1b/2a) levels by EI-1 alone and in combination with BILN-2061 or BMS-790052. HCV(1b/2a) persistently-infected cell cultures were treated with 5×EC_50_ concentrations of the indicated HCV inhibitors for 20 days (see Table 1 and Materials and Methods). HCV levels were normalized relative to the level of the DMSO control at each time point. This data is the average of 3 assays. Error bars represent standard deviation. Asterisks indicate statistically significant differences at day 20 from the DMSO day 20 time point (*t* test P≤0.05). DMSO (solid circles and solid line), EI-1 (pierced diamonds and dashed line), BILN-2061 (solid squares and dashed line), BMS-790052 (pierced squares and solid line), BILN-2061/EI-1 (diamonds and solid line),BMS-790052/EI-1 (solid hexagons and solid line), and BILN 2061/BMS-790052 (pierced diamonds and dotted line).

**Figure 4:**
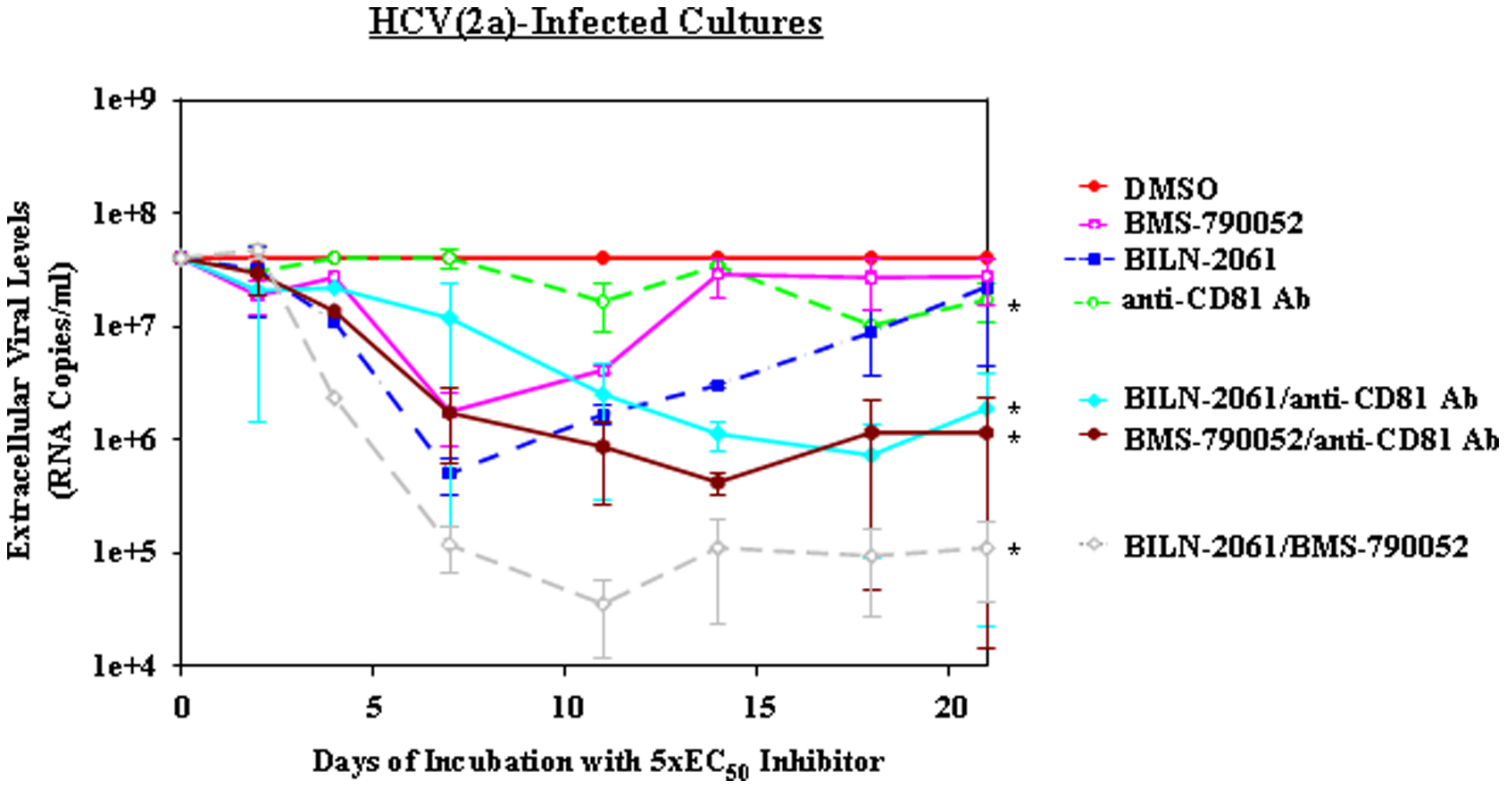
Reduction of HCV(2a) levels by anti-CD81 Ab alone and in combination with BILN-2061 or BMS-790052. HCV(2a) persistently-infected cell cultures were treated with 5×EC_50_ concentrations of the indicated HCV inhibitors for 21 days (see Table 1 and Materials and Methods). HCV levels were normalized relative to the level of the DMSO control at each time point. This data is the average of 3 assays. Error bars represent standard deviation. Asterisks indicate statistically significant differences at day 21 from the DMSO day 21 time point (*t* test P≤0.05). (A) DMSO (solid circles and solid line), anti-CD81 Ab (pierced circles and dashed line), BILN-2061 (solid squares and dashed-dot line), BMS-790052 (pierced squares and solid line), BILN-2061/anti-CD81 Ab (solid diamonds and solid line), BMS-790052/anti-CD81 Ab (solid hexagons and solid line), and BILN-2061/BMS-790052 (pierced diamonds and dashed line).

We sought to determine whether HCV entry inhibitor monotherapy could significantly reduce extracellular viral levels over time in HCV persistently-infected cultures. We first established stable persistently-infected cultures and then treated with either anti-CD81 antibody or Entry Inhibitor 1 (EI-1).These inhibitors were added to the cultures at 5×EC_50_ (“1×EC50” is defined as the concentration where the activity of the inhibitor is half maximal) (the 5×EC_50_ concentrations used were 2.5 µg/ml for anti-CD81 Ab and 170 nM for EI-1, see Table 1). During this treatment, the entry inhibitors anti-CD81 Ab and EI-1 slowly and very modestly reduced extracellular viral levels over time relative to DMSO-treated controls (Fig. 2, 3, and 4). With both anti-CD81 Ab and EI-1, we observed a <1 log_10_ RNA copies/ml reduction in extracellular viral RNA levels (HCV(1b/2a) and (2a)) over a 3 week time course. Importantly, neither anti-CD81 Ab, nor EI-1 exhibited cytotoxic effects at 5×EC_50_ concentrations. Anti-CD81 Ab and EI-1 exhibited CC_50_ values >5 µg/ml and >50 µM respectively in Huh7-Lunet-CD81 cells (Table 1). However, we observed through anti-NS5A staining after 3 weeks of entry inhibitor treatment a small reduction in the percentage of infected cells (Tables 2, 3, 4). It is likely that during entry inhibitor monotherapy extracellular HCV levels decreased very slowly over time because infected cells were slowly dying off. As we did not observe significant differences in the total number of cells in each well, a small fraction of uninfected cells that were protected from infection by the entry inhibitors likely proliferated to replace the lost cells.

**Table 1:** Summary of Antiviral Assay Results for the HCV Inhibitors Used in this Work.

Compound	EC_50_(nM)^a^ HCV(2a)	EC_50_(nM) HCV(1b/2a)^b^	CC_50_(nM)
**EI-1**	N.D.^c^	34	>50,000
**Anti-CD81 Ab**	0.5 µg/ml	0.5 µg/ml	>5 µg/ml
**BILN-2061**	106	74	>24,000
**BMS-790052**	0.02	0.01	16,635

a All values are reported in nM unless otherwise indicated.

b The HCV(1b/2a) chimeric virus expresses HCV(1b) envelope proteins and HCV(2a) replicative proteins [26].

c Not determined.

**Table 2:** Summary of HCV(1b/2a) Levels After 21 Days of Treatment +/− Anti-CD81 Ab.

Inhibitor(s)	Fold Extracellular RNA Suppression at Day 21	Estimated Percentage of Infected Cells at Day 21^a^
DMSO	1.0	++++
Anti-CD81 Ab	1.4	+++
BMS-790052	1.2	+++
BILN-2061	1.2	+++
BMS-790052/Anti-CD81 Ab	14	++
BILN-2061/Anti-CD81 Ab	554	+
BILN-2061/BMS-790052	512	+

a The percentage of infected cells was estimated after anti-NS5A Ab staining (see Materials and Methods).

**Table 3:** Summary of HCV(1b/2a) Levels After 20 Days of Treatment +/− EI-1.

Inhibitor(s)	Fold Extracellular RNA Suppression at Day 21	Estimated Percentage of Infected Cells at Day 20^a^
DMSO	1.0	++++
EI-1	3.1	+++
BMS-790052	1.2	+++
BILN-2061	1.2	+++
BMS-790052/EI-1	45	++
BILN-2061/EI-1	26	++
BILN-2061/BMS-790052	445	+

a The percentage of infected cells was estimated after anti-NS5A Ab staining (see Materials and Methods).

**Table 4:** Summary of HCV(2a) Levels After 21 Days of Treatment +/− Anti-CD81 Ab.

Inhibitor(s)	Fold Extracellular RNA Suppression at Day 21	Estimated Percentage of Infected Cells at Day 21^a^
DMSO	1.0	++++
Anti-CD81 Ab	2.4	+++
BMS-790052	1.5	+++
BILN-2061	1.8	+++
BMS-790052/Anti-CD81 Ab	35	++
BILN-2061/Anti-CD81 Ab	21	++
BILN-2061/BMS-790052	363	+

a The percentage of infected cells was estimated after anti-NS5A Ab staining (see Materials and Methods).

### HCV Replication Inhibitor Monotherapy Reduced Viral Levels in a Biphasic Manner Over Time in Persistently-infected Cultures

We also investigated whether HCV replication inhibitor monotherapy reduced extracellular viral levels over time in these cultures. We treated HCV persistently-infected cultures with 5×EC_50_ concentrations (see Table 1) of the NS3-4A protease inhibitor BILN-2061 or with the NS5A inhibitor BMS-790052. We observed that both the NS3-4A protease inhibitor and the NS5A inhibitor reduced HCV(1b/2a) (Fig. 2 and 3) and HCV(2a) (Fig. 4) extracellular levels (1–2 log_10_ RNA copies/ml) in a rapid, biphasic manner during the initial 7 to 10 days of treatment. After this initial reduction, in all cases, extracellular viral levels started rising again. This rise in the extracellular viral levels can be attributed to the appearance of resistance mutations. Through clonal sequencing, we found that the previously reported resistance mutations to each inhibitor appeared by the end of each time course (Table 5). D168N in NS3 was observed after protease inhibitor BILN-2061 treatment and NS5A Y93H was observed after NS5A inhibitor BMS-790052 treatment. These resistance mutations have been previously reported using these inhibitors [4], [32], [33]. This observed rapid, biphasic reduction in viral levels caused by replication inhibitor montherapy was predicted by viral dynamic modelling [22] and has been observed in clinical trials [34]. Furthermore, our clonal sequencing results suggested that resistance mutations against the replication inhibitors were acquired over time by members of the viral population.

**Table 5:** Summary of the Resistance Mutations Observed After 3 weeks of HCV Inhibitor Treatment.

Compound	HCV(2a)^a^	HCV(1b/2a)a,b
EI-1	N.D.^c^	E2 V719G/L
Anti-CD81 Ab	E2 N430A/E, D431K, S432L, I438V, A439C/T, S440Q	E2 C429I/Q, N430P/S, A439E
BILN-2061	NS3 D168N	NS3 D168N
BMS-790052	NS5A Y93H	NS5A Y93H

a These resistance mutations were observed in 1/5 sequenced clones.

b The HCV(1b/2a) chimeric virus expresses HCV(1b) envelope proteins and HCV(2a) replicative proteins [26].

c Not determined.

Besides measuring a reduction in extracellular HCV RNA levels as a measure of viral inhibition, we also measured the percentage of infected cells after inhibitor treatments. We observed that at the end of each time course the relative differences in the percentages of infected cells per well corresponded roughly with the HCV RNA levels. Specifically, we observed only a slight decrease in the percentage of infected cells after 3 weeks of treatment with the replication inhibitors relative to the DMSO control (Tables 2, 3, 4). This corresponded with the rebound in extracellular HCV RNA levels also observed after 3 weeks (Fig. 2, 3, and 4).

### Combination Therapy with an HCV Entry Inhibitor and an HCV Replication Inhibitor Promoted a Greater and More Prolonged Viral Reduction than Monotherapy

Because HCV entry and replication inhibitors hinder different aspects of the viral lifecycle, we asked if combining an entry inhibitor with a replication inhibitor would reduce viral levels to a greater extent than treatment with either inhibitor alone. We observed that the combination of the entry inhibitor anti-CD81 Ab with the NS3-4A protease inhibitor BILN-2061 or with NS5A inhibitor BMS-790052 led to greater reductions in HCV(1b/2a) levels over time compared to monotherapy treatment in all cases (Fig. 2). Replication inhibitor monotherapy and replication inhibitor/anti-CD81 Ab combination therapies both reduced extracellular HCV(1b/2a) levels 1–2 log_10_ RNA copies/ml after 7–10 days of treatment (Fig. 2). However, extracellular HCV(1b/2a) levels were maintained at low levels on day 21 for the replication inhibitor/anti-CD81 Ab combinations, whereas all replication inhibitor monotherapy resulted in increases in extracellular viral levels (compared to nadirs) at this time (Fig. 2). For the replication inhibitor/anti-CD81 Ab combinations in HCV(1b/2a), there was still a 1–2 log_10_ RNA copies/ml reduction at 21 days (Fig. 2). In terms of fold reduction for RNA levels, the BMS-790052/anti-CD81 Ab and BILN-2061/anti-CD81 Ab combinations reduced HCV(1b/2a) RNA levels 14-fold and 554-fold respectively relative to the DMSO control at day 21 (Table 2). Thus, the BILN-2061/anti-CD81 Ab combination was clearly the most potent replication inhibitor/entry inhibitor combination for maintaining low HCV RNA levels over an extended period of time. This conclusion was further supported by the percentage of infected cells at day 21 for these combinations (Table 2 and Fig. 1A).

Besides testing the entry inhibitor anti-CD81 Ab in combination with replication inhibitors in HCV(1b/2a), we also tested EI-1 in combination with replication inhibitors. When we treated the HCV(1b/2a) cultures with the protease inhibitor BILN-2061 or NS5A inhibitor BMS-790052 combined with EI-1, we observed that viral levels were reduced up to 2.5 log_10_ RNA copies/mls over 14 days compared to a 1–2 log_10_ RNA copies/ml reduction during replication inhibitor monotherapy (Fig. 3). A much slower viral rebound was observed in the HCV(1b/2a) case for the replication inhibitor/EI-1 combinations compared to replication inhibitor monotherapy (Fig. 3). At day 20, the BMS-790052/EI-1 combination maintained RNA levels that were 45-fold lower than the DMSO-treated control and the BILN-2061/EI-1 combination maintained RNA levels that were 26 fold lower than the DMSO-treated control (Table 3). The relative differences in the percentage of infected cells reflected these results when compared to the DMSO-treated control in each case (Table 3). Together, these data suggested that both the BMS-790052/EI-1 and BILN-2061/EI-1 combinations maintained a strong reduction in HCV levels and reduced the percentage of infected cells after 20 days of treatment relative to the DMSO-treated control. Based upon the day 20 HCV RNA levels and the estimated percentage of infected cells in each case at that time, the BMS-790052/EI-1 and BILN-2061/EI-1 combinations were roughly equipotent over an extended time period.

In addition to studying replication/entry inhibitor combinations in HCV(1b/2a), we performed a similar set of experiments with HCV(2a). As with HCV(1b/2a), we observed that monotherapy with the protease inhibitor BILN-2061 and the NS5A inhibitor BMS-790052 led to a 1–2 log10 RNA copies/ml reduction during the first 7 days or so followed by a rebound in extracellular RNA levels (Fig. 4). In the cases where the replication inhibitors were combined with the entry inhibitor anti-CD81 Ab, we observed a 2 log_10_ RNA copies/ml reduction. Similarly to the HCV(1b/2a) experiments, the reduction in extracellular HCV(2a) RNA levels was prolonged for the duration of the time course when entry and replication inhibitors were combined (Fig. 4). BMS-790052/anti-CD81 Ab and BILN-2061/anti-CD81 Ab combinations caused a 35-fold and 21-fold reduction respectively in RNA levels at day 21 relative to the DMSO-treated control (Table 4). These results were also reflected by the differences in the relative percentages of infected cells at day 21 (Table 4). These data suggested that both of these replication inhibitor/anti-CD81 Ab combinations were similarly potent at maintaining low HCV levels over a 3-week time course.

Besides measuring extracellular viral reductions resulting from combination treatment with an entry and replication inhibitors, we also investigated whether the combination of two replication inhibitors targeting different aspects of HCV replication could comparably reduce viral levels. Thus, we combined the NS3–4A protease inhibitor BILN-2061 with the NS5A inhibitor BMS-790052 and quantified viral levels over time. In HCV(1b/2a)-infected cells, we observed that the replication inhibitor combination of BILN-2061/BMS-790052 caused a faster reduction in viral levels over 14 days (3 log_10_ RNA copies/ml) than the replication/entry inhibitor combinations (2 log_10_ RNA copies/ml) (Fig. 2 and Fig. 3). The combination of these two replication inhibitors yielded a 512-fold and 445-fold reduction in RNA levels at the final time point relative to the DMSO control (Tables 2 and 3). Furthermore, the combination of the two replication inhibitors yielded the lowest levels of infected cells after extended treatment out of all of the inhibitor treatments studied here, except for the BILN-2061/anti-CD81 Ab case (Tables 2 and 3). Only the combination of BILN-2061/anti-CD81 Ab yielded similar results with regard to RNA levels and percentage of infected cells at day 21, though notably the rate of reduction was slower than with BILN-2061/BMS-790052 (Fig. 2 and Table 2). In the HCV(2a) case, the BILN-2061/BMS-790052 combination caused viral levels to be reduced 3 log_10_ RNA copies/ml over time before plateauing at day 14 (Fig. 4). This result was in contrast to the combination therapy with replication/entry inhibitors which caused HCV(2a) levels to only be reduced 2 log_10_ RNA copies/mls over 21 days (Fig. 4). In addition, the combination of the two replication inhibitors maintained the lowest percentage of HCV(2a) infected cells at day 21 (Table 4). Together, these results suggested that the BILN-2061/BMS-790052 replication inhibitor combination exhibited greater and more prolonged antiviral effects than EI-1 plus either replication inhibitor in HCV(1b/2a) or than anti-CD81 Ab plus either replication inhibitor in HCV(2a). However, BILN-2061/anti-CD81 Ab treatment promoted similar HCV(1b/2a) levels as BILN-2061/BMS-790052 after 3 weeks of treatment, though BILN-2061/anti-CD81 Ab reduced the viral levels more slowly than BILN-2061/BMS-790052.

For most of the treatment cases studied, we checked if resistance mutations had arisen by day 21 using clonal sequencing (Table 5). When anti-CD81 Ab was used alone or in combination with replication inhibitors, we identified the E2 domain Ia mutations N430A/E, D431K, S432L, I438V, A439C/T, and S440Q among others (Table 5) similar to those previously reported [11]. For EI-1 alone or in combination with replication inhibitors, the E2 transmembrane domain mutations V719G/L were observed as have been reported by others [2] (Table 5). Also, in cases where entry inhibitors and replication inhibitors were combined, we found NS3 D168N after treating with BILN-2061 [4] and NS5A Y93H [33] after treating with BMS-790052 (Table 5). Interestingly, none of these mutations were observed using population sequencing, suggesting that only a subset of each viral population had acquired the resistance mutations at the time of sampling.

## Discussion

Here we showed that HCV entry inhibitor monotherapy only slowly reduced extracellular viral levels in persistently-infected cell cultures where most of the cells are infected. These results suggest that entry inhibitor monotherapies will only have a modest impact on serum HCV RNA in patients who have only minimal viral spreading at the time of treatment. Moreover, these findings are in agreement with recent reports that HCV entry inhibitor monotherapy with JTK-652 [35], and ITX-5061 [36] had no effect on patient serum HCV RNA. Nevertheless, our model system is unlikely to closely mimic the dynamics of HCV infection in the liver. For example, the results generated with our persistently-infected cell culture model do not serve as a model for HCV patients whose infection is rapidly spreading. Entry inhibitor monotherapy would likely potently inhibit serum HCV RNA in patients whose infection is rapidly spreading. In our assays, entry inhibitor treatments likely produced a slow decline in viral levels because HCV-infected cells continually turn over due to apoptotic mechanisms [37]. In addition, multiple rounds of infection of naïve cells appear to be required to sustain HCV infection in cell culture and presumably *in vivo* [38]. Consistent with these findings, we observed a small decrease in the percentage of infected cells as well as in extracellular HCV RNA levels during entry inhibitor monotherapy.

In addition to showing that HCV entry inhibitors only provided a slow reduction of viral levels in persistently-infected cell cultures with little viral spreading, we demonstrated that replication inhibitors provided a rapid reduction in viral levels in this model system followed by rebound. Moreover, entry/replication inhibitor treatment prolonged lower viral levels after 3 weeks than either monotherapy. These results were most likely due to a delay in the emergence of resistance to one or both of the inhibitors.

Differences in genetic resistance barriers and viral fitness likely explain why specific combinations of entry and replication inhibitors proved to be more potent than others. We observed that in the HCV(1b/2a) case the BILN-2061/anti-CD81 Ab combination exhibited a more potent antiviral response than BMS-790052/anti-CD81 Ab or BILN-2061/EI-1 (Fig. 2 and 3). These results suggest that there is a higher genetic resistance barrier for the BILN-2061/anti-CD81 Ab combination in HCV(1b/2a) than for the other cases. This is likely the case for two reasons. First, multiple mutations in E2 domain Ia are required to confer resistance to anti-CD81 Ab [11], while a single E2 transmembrane domain mutation can grant resistance against EI-1 [2]. Second, the combination of E2(1b)/NS3(2a) mutations needed to exhibit resistance against anti-CD81 Ab/BILN-2061 may be less fit than the combination of required resistance mutations in E2(1b)/NS5A(2a) needed to exhibit resistance against anti-CD81 Ab/BMS-790052.

Notably, BILN-2061/anti-CD81 Ab treatment in the HCV(2a) case was not as potent as in HCV(1b/2a) (Fig. 4 and Fig. 2). Rather BILN-2061/anti-CD81 Ab treatment in HCV(2a) was more similar to BMS-790052/anti-CD81 Ab treatment in HCV(2a). It is likely that the resistance mutations in E2(2a)/NS3(2a) and in E2(2a)/NS5A(2a) were more readily acquired and reduced viral fitness less than in the E2(1b)/NS3(2a) case.

Interestingly the combination of two replication inhibitors strongly and rapidly decreased viral levels over time for both HCV(2a) and HCV(1b/2a) (Fig. 2–4). The fact that the two inhibitors that were combined (BILN-2061 and BMS-790052) target different HCV proteins (NS3–4A and NS5A respectively), meant that a higher resistance barrier was established when combined. Because RNA replication was being inhibited by two different mechanisms, the acquisition of resistance mutations was severely slowed. The BILN-2061/BMS-790052 combination therapy promoted the greatest reduction in HCV(2a) levels after 3 weeks out of the tested combinations (Fig. 4) and one of the greatest reductions in HCV(1b/2a) levels after 3 weeks along with the BILN-2061/anti-CD81 Ab combination (Fig. 2). Thus, BILN-2061/BMS-790052 in HCV(2a) along with BILN-2061/anti-CD81 Ab in HCV(1b/2a) likely provided the greatest resistance barriers relative to the other combinations tested. However, the BILN-2061/anti-CD81 Ab combination did not reduce the viral RNA levels as rapidly as the BILN-2061/BMS-790052 combination. With the BILN-2061/anti-CD81 Ab combination, anti-CD81 Ab could prevent re-infection of cells cured by BILN-2061, but could not accelerate the reduction in viral RNA levels. Overall, our results suggest that the right combination of entry/replication inhibitor could provide a powerful addition to an HCV treatment regimen, but that the best combinations may differ depending upon the HCV genotype. Ideally, regimens will be identified that provide robust efficacy against all genotypes in order to simplify the treatment of chronic HCV.

## References

[1] Herker E, Harris C, Hernandez C, Carpentier A, Kaehlcke K, et al (2010) Efficient hepatitis C virus particle formation requires diacylglycerol acyltransferase-1. Nat Med 16: 1295–1298. 20935628 10.1038/nm.2238 PMC3431199

[2] Baldick CJ, Wichroski MJ, Pendri A, Walsh AW, Fang J, et al (2010) A novel small molecule inhibitor of hepatitis C virus entry. PLoS Pathog 6: e1001086. 20838466 10.1371/journal.ppat.1001086 PMC2936744

[3] Fridell RA, Wang C, Sun JH, O’Boyle DR, 2nd, Nower P, et al (2011) Genotypic and phenotypic analysis of variants resistant to hepatitis C virus nonstructural protein 5A replication complex inhibitor BMS-790052 in humans: in vitro and in vivo correlations. Hepatology 54: 1924–1935. 21809362 10.1002/hep.24594

[4] Lenz O, Verbinnen T, Lin TI, Vijgen L, Cummings MD, et al (2010) In vitro resistance profile of the hepatitis C virus NS3/4A protease inhibitor TMC435. Antimicrob Agents Chemother 54: 1878–1887. 20176898 10.1128/AAC.01452-09 PMC2863659

[5] Goldwasser J, Cohen PY, Lin W, Kitsberg D, Balaguer P, et al (2011) Naringenin inhibits the assembly and long-term production of infectious hepatitis C virus particles through a PPAR-mediated mechanism. J Hepatol 55: 963–971. 21354229 10.1016/j.jhep.2011.02.011 PMC3197749

[6] Dorner M, Horwitz JA, Robbins JB, Barry WT, Feng Q, et al (2011) A genetically humanized mouse model for hepatitis C virus infection. Nature 474: 208–211. 21654804 10.1038/nature10168 PMC3159410

[7] Sainz B Jr, Barretto N, Martin DN, Hiraga N, Imamura M, et al (2012) Identification of the Niemann-Pick C1-like 1 cholesterol absorption receptor as a new hepatitis C virus entry factor. Nat Med 18: 281–285. 22231557 10.1038/nm.2581 PMC3530957

[8] Mittapalli GK, Zhao F, Jackson A, Gao H, Lee H, et al.. (2012) Discovery of ITX 4520: A highly potent orally bioavailable hepatitis C virus entry inhibitor. Bioorg Med Chem Lett. 10.1016/j.bmcl.2012.06.038 22784640

[9] Zhu H, Wong-Staal F, Lee H, Syder A, McKelvy J, et al (2012) Evaluation of ITX 5061, a scavenger receptor B1 antagonist: resistance selection and activity in combination with other hepatitis C virus antivirals. J Infect Dis 205: 656–662. 22279172 10.1093/infdis/jir802 PMC3266130

[10] Ciesek S, von Hahn T, Colpitts CC, Schang LM, Friesland M, et al (2011) The green tea polyphenol, epigallocatechin-3-gallate, inhibits hepatitis C virus entry. Hepatology 54: 1947–1955. 21837753 10.1002/hep.24610

[11] Keck ZY, Saha A, Xia J, Wang Y, Lau P, et al (2011) Mapping a region of hepatitis C virus E2 that is responsible for escape from neutralizing antibodies and a core CD81-binding region that does not tolerate neutralization escape mutations. J Virol 85: 10451–10463. 21813602 10.1128/JVI.05259-11 PMC3187491

[12] Barth H, Schafer C, Adah MI, Zhang F, Linhardt RJ, et al (2003) Cellular binding of hepatitis C virus envelope glycoprotein E2 requires cell surface heparan sulfate. J Biol Chem 278: 41003–41012. 12867431 10.1074/jbc.M302267200

[13] Pileri P, Uematsu Y, Campagnoli S, Galli G, Falugi F, et al (1998) Binding of hepatitis C virus to CD81. Science 282: 938–941. 9794763 10.1126/science.282.5390.938

[14] Scarselli E, Ansuini H, Cerino R, Roccasecca RM, Acali S, et al (2002) The human scavenger receptor class B type I is a novel candidate receptor for the hepatitis C virus. EMBO J 21: 5017–5025. 12356718 10.1093/emboj/cdf529 PMC129051

[15] Evans MJ, von Hahn T, Tscherne DM, Syder AJ, Panis M, et al (2007) Claudin-1 is a hepatitis C virus co-receptor required for a late step in entry. Nature 446: 801–805. 17325668 10.1038/nature05654

[16] Ploss A, Evans MJ, Gaysinskaya VA, Panis M, You H, et al (2009) Human occludin is a hepatitis C virus entry factor required for infection of mouse cells. Nature 457: 882–886. 19182773 10.1038/nature07684 PMC2762424

[17] Tscherne DM, Jones CT, Evans MJ, Lindenbach BD, McKeating JA, et al (2006) Time- and temperature-dependent activation of hepatitis C virus for low-pH-triggered entry. J Virol 80: 1734–1741. 16439530 10.1128/JVI.80.4.1734-1741.2006 PMC1367161

[18] Lupberger J, Zeisel MB, Xiao F, Thumann C, Fofana I, et al (2011) EGFR and EphA2 are host factors for hepatitis C virus entry and possible targets for antiviral therapy. Nat Med 17: 589–595. 21516087 10.1038/nm.2341 PMC3938446

[19] Meertens L, Bertaux C, Dragic T (2006) Hepatitis C virus entry requires a critical postinternalization step and delivery to early endosomes via clathrin-coated vesicles. J Virol 80: 11571–11578. 17005647 10.1128/JVI.01717-06 PMC1642584

[20] Cormier EG, Tsamis F, Kajumo F, Durso RJ, Gardner JP, et al (2004) CD81 is an entry coreceptor for hepatitis C virus. Proc Natl Acad Sci U S A 101: 7270–7274. 15123813 10.1073/pnas.0402253101 PMC409908

[21] Koutsoudakis G, Kaul A, Steinmann E, Kallis S, Lohmann V, et al (2006) Characterization of the early steps of hepatitis C virus infection by using luciferase reporter viruses. J Virol 80: 5308–5320. 16699011 10.1128/JVI.02460-05 PMC1472176

[22] Neumann AU, Lam NP, Dahari H, Gretch DR, Wiley TE, et al (1998) Hepatitis C viral dynamics in vivo and the antiviral efficacy of interferon-alpha therapy. Science 282: 103–107. 9756471 10.1126/science.282.5386.103

[23] Dvory-Sobol H, Wong KA, Ku KS, Bae A, Lawitz EJ, et al.. (2012) Characterization of Resistance to the Protease Inhibitor GS-9451 in Hepatitis C Virus-Infected Patients. Antimicrob Agents Chemother. 10.1128/AAC.00780-12 PMC3457362 22869562

[24] Powdrill MH, Tchesnokov EP, Kozak RA, Russell RS, Martin R, et al (2011) Contribution of a mutational bias in hepatitis C virus replication to the genetic barrier in the development of drug resistance. Proc Natl Acad Sci U S A 108: 20509–20513. 22135458 10.1073/pnas.1105797108 PMC3251051

[25] Pelosi LA, Voss S, Liu M, Gao M, Lemm JA (2012) Effect on HCV Replication by Combinations of Direct Acting Antivirals Including NS5A Inhibitor Daclatasvir. Antimicrob Agents Chemother. 10.1128/AAC.01209-12 PMC3457360 22850513

[26] Chan K, Cheng G, Beran RK, Yang H, Appleby TC, et al (2012) An adaptive mutation in NS2 is essential for efficient production of infectious 1b/2a chimeric hepatitis C virus in cell culture. Virology 422: 224–234. 22099378 10.1016/j.virol.2011.10.022

[27] Schultz B, Yang H, Delaney WEt (2011) Biochemical evaluation of HCV NS3 protease inhibitors. Curr Protoc Pharmacol Chapter 13: Unit13B 17. 10.1002/0471141755.ph13b07s54 21898332

[28] Sainz B Jr, Chisari FV (2006) Production of infectious hepatitis C virus by well-differentiated, growth-arrested human hepatoma-derived cells. J Virol 80: 10253–10257. 17005703 10.1128/JVI.01059-06 PMC1617281

[29] Beran RK, Sharma R, Corsa AC, Tian Y, Golde J, et al (2012) Cellular growth kinetics distinguish a cyclophilin inhibitor from an HSP90 inhibitor as a selective inhibitor of hepatitis C virus. PLoS One 7: e30286. 22347373 10.1371/journal.pone.0030286 PMC3275588

[30] Pokrovskii MV, Bush CO, Beran RK, Robinson MF, Cheng G, et al (2011) Novel mutations in a tissue culture-adapted hepatitis C virus strain improve infectious-virus stability and markedly enhance infection kinetics. J Virol 85: 3978–3985. 21289124 10.1128/JVI.01760-10 PMC3126117

[31] Bauhofer O, Ruggieri A, Schmid B, Schirmacher P, Bartenschlager R (2012) Persistence of HCV in quiescent hepatic cells under conditions of an interferon-induced antiviral response. Gastroenterology 143: 429–438 e428. 10.1053/j.gastro.2012.04.018 22522091

[32] Summa V, Ludmerer SW, McCauley JA, Fandozzi C, Burlein C, et al.. (2012) MK-5172, a selective inhibitor of Hepatitis C Virus NS3/4a protease with broad activity across genotypes and resistant variants. Antimicrob Agents Chemother. 10.1128/AAC.00324-12 PMC3421554 22615282

[33] Fridell RA, Qiu D, Valera L, Wang C, Rose RE, et al (2011) Distinct functions of NS5A in hepatitis C virus RNA replication uncovered by studies with the NS5A inhibitor BMS-790052. J Virol 85: 7312–7320. 21593143 10.1128/JVI.00253-11 PMC3126594

[34] Dvory-Sobol H, Wong KA, Ku KS, Bae A, Lawitz EJ, et al (2012) Characterization of resistance to the protease inhibitor GS-9451 in hepatitis C virus-infected patients. Antimicrob Agents Chemother 56: 5289–5295. 22869562 10.1128/AAC.00780-12 PMC3457362

[35] de Bruijne J, Bergmann JF, Weegink CJ, van Nieuwkerk CM, de Knegt RJ, et al (2010) Safety and antiviral activity of JTK-652: a novel HCV infection inhibitor. Antivir Ther 15: 765–773. 20710058 10.3851/IMP1606

[36] Sulkowski M, Kang M, Matining R, Wyles D, Johnson V, et al.. (2013) A Randomized, Double-Blind Phase 1b Study to Assess the Safety and Activity of the HCV Entry Inhibitor ITX-5061 in Treatment-Naive HCV Mono-Infected Adults: A5277 March 3, 2013; CROI Atlanta, GA, USA. 10.1093/infdis/jit503 PMC3923538 24041792

[37] Deng L, Adachi T, Kitayama K, Bungyoku Y, Kitazawa S, et al (2008) Hepatitis C virus infection induces apoptosis through a Bax-triggered, mitochondrion-mediated, caspase 3-dependent pathway. J Virol 82: 10375–10385. 18768989 10.1128/JVI.00395-08 PMC2573220

[38] Coburn GA, Fisch DN, Moorji SM, de Muys JM, Murga JD, et al (2012) Novel small-molecule inhibitors of hepatitis C virus entry block viral spread and promote viral clearance in cell culture. PLoS One 7: e35351. 22545104 10.1371/journal.pone.0035351 PMC3335862

